# Mapping insecticide resistance and characterization of resistance mechanisms in *Anopheles arabiensis* (Diptera: Culicidae) in Ethiopia

**DOI:** 10.1186/s13071-017-2342-y

**Published:** 2017-09-02

**Authors:** Eba Alemayehu, Abebe Asale, Kasahun Eba, Kefelegn Getahun, Kora Tushune, Astrid Bryon, Evangelia Morou, John Vontas, Thomas Van Leeuwen, Luc Duchateau, Delenasaw Yewhalaw

**Affiliations:** 1 Department of Biology, College of Natural Sciences, Jimma University, Jimma, Ethiopia; 2 Tropical and Infectious Diseases Research Center, Jimma University, Jimma, Ethiopia; 3 Department of Comparative Physiology and Biometrics, University of Ghent, Ghent, Belgium; 4 Department of Geography and Environmental Studies, Jimma University, Jimma, Ethiopia; 5 Department of Health Services Management, College of Health Sciences, Jimma University, Jimma, Ethiopia; 6 Department of Crop Protection, Ghent University, Ghent, Belgium; 7 Department of Biology, University of Crete, Heraklion, Greece; 8 Institute of Molecular Biology and Biotechnology, Foundation for Research and Technology-Hellas, Heraklion, Greece; 9 Department of Crop Science, Pesticide Science Lab, Agricultural University of Athens, Athens, Greece; 10 Institute for Biodiversity and Ecosystem Dynamics, University of Amsterdam, Amsterdam, The Netherlands; 11 Department of Medical Laboratory Sciences and Pathology, College of Health Sciences, Jimma University, Jimma, Ethiopia

**Keywords:** Malaria, Insecticide resistance, *Anopheles arabiensis*, Resistance mechanisms, Vector control, Ethiopia

## Abstract

**Background:**

The emergence and spread of insecticide resistance in the major African malaria vectors *Anopheles gambiae* (*s.s*.) and *An*. *arabiensis* may compromise the current vector control interventions and threatens the global malaria control and elimination efforts.

**Methods:**

Insecticide resistance was monitored in several study sites in Ethiopia from 2013 to 2015 using papers impregnated with discriminating concentrations of DDT, deltamethrin, bendiocarb, propoxur, malathion, fenitrothion and pirimiphos-methyl, following the WHO insecticide susceptibility test procedure. Mosquitoes sampled from different localities for WHO bioassay were morphologically identified as *An*. *gambiae* (*s.l*.) using standard taxonomic keys. Samples were identified to species using species-specific polymerase chain reaction (PCR) and screened for the presence of target site mutations L1014F, L1014S and N1575Y in the voltage gated sodium channel (*VGSC*) gene and G119S in the acethylcholinesterase (*AChE*) gene using allele-specific PCR. Biochemical assays were performed to assess elevated levels of acetylcholinesterases, carboxylcholinesterases, glutathione-S-transferases (GSTs) and cytochrome P450s monooxygenases in wild populations of *An*. *arabiensis*, compared to the fully susceptible Sekoru *An*. *arabiensis* laboratory strain.

**Results:**

Populations of *An*. *arabiensis* were resistant to DDT and deltamethrin but were susceptible to fenitrothion in all the study sites. Reduced susceptibility to malathion, pirimiphos-methyl, propoxur and bendiocarb was observed in some of the study sites. Knockdown resistance (*kdr* L1014F) was detected in all mosquito populations with allele frequency ranging from 42 to 91%. Elevated levels of glutathione-S-transferases (GSTs) were detected in some of the mosquito populations. However, no elevated levels of monooxygenases and esterases were detected in any of the populations assessed.

**Conclusions:**

*Anopheles arabiensis* populations from all surveyed sites in Ethiopia exhibited resistance against DDT and pyrethroids. Moreover, some mosquito populations exhibited resistance to propoxur and possible resistance to bendiocarb. Target site mutation *kdr* L1014F was detected in all mosquito populations while elevated levels of glutathione-S-transferases (GSTs) was detected in some mosquito populations. The reduced susceptibility of *An*. *arabiensis* to propoxur and bendiocarb, which are currently used for indoor residual spraying (IRS) in Ethiopia, calls for continuous resistance monitoring, in order to plan and implement evidence based insecticide resistance management.

**Electronic supplementary material:**

The online version of this article (10.1186/s13071-017-2342-y) contains supplementary material, which is available to authorized users.

## Background

Malaria is endemic in 97 countries, mostly in sub-Saharan Africa, and over 200 million people worldwide are estimated to be infected, with over half a million deaths worldwide [1, 2]. Globally, there are 472 described species, and over 50 unnamed members of species complexes, in the genus *Anopheles* [3], of which 70 species are known to be major malaria vectors [4]. Of the over 140 described species of the genus *Anopheles* in Africa, eight species are known to be efficient vectors of malaria [5, 6]. *Anopheles gambiae* Giles (*s.s*.), *An*. *coluzzii* Coetzee & Wilkerson, *An*. *arabiensis* Patton and *An*. *funestus* Giles are the most important and widely distributed vectors in the region [5, 6].

Vector control is one of the main approaches to combat malaria. Several interventions are being implemented by malaria endemic countries, of which chemical insecticides remain the mainstay [7, 8]. The contribution of indoor residual spraying (IRS) and long-lasting insecticidal nets (LLINs) are instrumental in protecting people from malaria. However, the emergence and spread of insecticide resistance in the major African malaria vectors, *An*. *gambiae* (*s.s*.) and *An*. *arabiensis*, may compromise the current IRS or LLINs based malaria control interventions and thus threaten malaria control and elimination efforts [1, 9–18]. Moreover, the poor understanding of the geographical distribution of the underlying insecticide resistance mechanisms makes it difficult to plan and implement efficient insecticide resistance management strategies, insecticide choice and insecticide use in time and space [19]. In most cases, resistance is attributed to two major mechanisms: (i) target-site insensitivity, where mutations in the target-site of the insecticide alter binding; and (ii) metabolic-based resistance, where the insecticide is degraded, sequestered or transported/excreted out of the cell before it can bind to its target [19].

In many malaria endemic African countries, both target-site and metabolic resistance mechanisms have been reported in malaria vectors. Target site resistance to pyrethroids and DDT is associated with mutations in the voltage-gated sodium channel in mosquito nerve membranes [20–22], which cause knockdown resistance (*kdr*). In *Anopheles*, this involves the substitution of leucine (TTA) to phenylalanine (TTT) (*kdr* L1014F) or to serine (TCA) (*kdr* L1014S) [20, 21]. In addition, substitution of asparagine to tyrosine (N1575Y) is associated with resistance in *An*. *gambiae* [23]. There is also an acetylcholinesterase gene (ace-1^R^) mutation, where a glycine (GGC) is substituted to a serine (AGC) which confers resistance to organophosphates and carbamates [24].

Metabolic resistance mediated by detoxifying enzymes also plays a significant role in insecticide resistance in malaria vectors [25]. Elevated levels of cytochrome P450 monooxygenases (P450s), carboxylcholinesterases (CCEs) and glutathione S-transferases (GSTs) in mosquitoes may confer resistance to different classes of insecticides. These enzymes detoxify or sequester insecticides before reaching the target site of action. The role of detoxification based resistance alone or in combination with target-site resistance in the major malaria vectors has been reported in scientific literature [26–30].

In Ethiopia, over 60% of the population lives in malarious areas [31]. *Plasmodium vivax* and *P*. *falciparum* are responsible for the majority of malaria cases and both species coexist in the country with a prevalence that varies according to season and locality. In most parts of the country, malaria transmission is seasonal and unstable, which leads to outbreaks or cyclic epidemics [1, 32]. Forty two species of *Anopheles* have been reported in Ethiopia and, of these, *An*. *arabiensis*, a member of the *An*. *gambiae* complex, is the main malaria vector in the country. Secondary vectors, such as *An*. *funestus* group, *An*. *pharoensis* and *An*. *nili*, occur more sporadically and with limited distribution in the country [32].

The number of malaria cases has declined in Ethiopia since 2006 due to a high coverage of IRS and scaling up of LLINs [1, 33]. This initiated the development of the national malaria elimination road map by the national malaria control and elimination program to eliminate malaria from Ethiopia by 2030 [34]. However, the emergence and spread of insecticide resistance in *An*. *arabiensis* could threaten such elimination efforts in the country [1, 12–17].

In Ethiopia, target site resistance mechanism in populations of *An*. *arabiensis* was first reported from areas around the Gilgel Gibe hydro-electric dam, southwestern Ethiopia. The *kdr* allele frequency of the L1014F mutation in the Gilgel Gibe region was the highest ever reported in *An*. *arabiensis* [12]. Subsequent studies have also documented the same mutation in this species in other parts of the country [15–17]. However, the frequency of *kdr* allele in some other malarious areas of the country is not yet documented, as only few and scattered reports are available. Moreover, it is unclear whether mechanisms, other than *kdr*, are involved in conferring resistance to insecticides in populations of *An*. *arabiensis* from Ethiopia. Thus, this study aimed to investigate the distribution of insecticide resistance in some selected malarious areas and characterize target site and metabolic resistance mechanisms in malaria vectors in Ethiopia.

## Methods

### Study sites and mosquito sampling

**Fig. 1:**
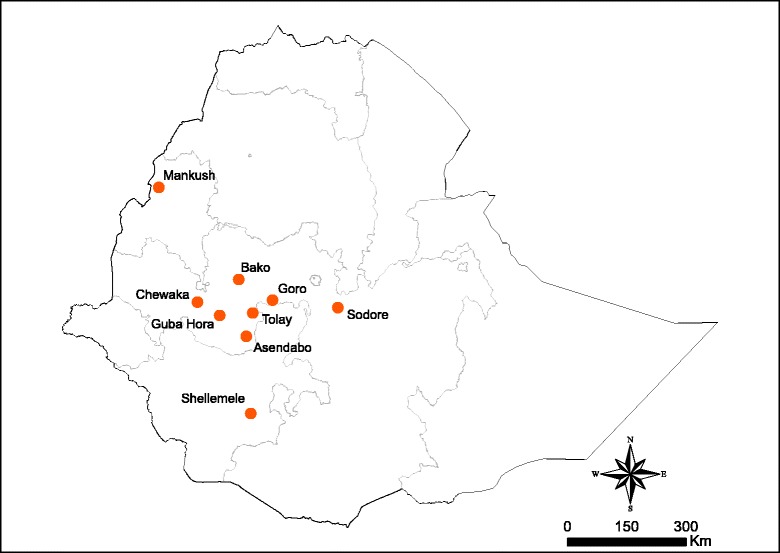
Map of Ethiopia showing the study sites

Nine study sites were selected from malarious regions of Ethiopia (Fig. 1). The sites were selected to represent the most important malaria endemic areas from central, western, south-western and southern parts of the country. The study sites were Mankush, Chewaka, Tolay, Asendabo, Bako, Sodore, Shellemele, Goro and Guba Hora. Insecticide resistance was monitored for three years (2013–2015) in Mankush, Chewaka and Shellemele, whereas in Asendabo, Tolay and Sodore resistance was monitored for two years (2014–2015). The insecticide resistance survey in Bako site was conducted in 2013 while in Goro and Guba Hora sites the resistance survey was conducted in 2015. In each study site, anopheline mosquito larvae were collected during the wet season (July–September) by dipping from a range of breeding sites: road puddles, brick pits, pools, marshes, streams, ditches, pits dug for plastering traditional tukuls, and pits dug for pot making. The collected larvae were reared to adults in the respective study sites under field-testing conditions. Temperature and relative humidity in all field-testing rooms in each study sites were within the range of 25 ± 2 °C and 80 ± 10%, respectively. The larvae were fed with dog biscuit and brewery yeast [35]. Mosquitoes were initially identified morphologically as *An*. *gambiae* (*s.l*.) using a taxonomic key [5].

### Insecticide susceptibility tests

Non blood-fed adult female mosquitoes (2–3 day-old), were exposed to insecticide impregnated papers with discriminating concentrations of DDT (4%), malathion (5%), deltamethrin (0.05%), bendiocarb (0.1%), pirimiphos-methyl (0.25%), fenitrothion (1%) and propoxur (0.1%), following the WHO insecticide susceptibility test procedure [36]. Insecticides were selected based on their current operational significance in the national malaria control program. Pirimiphos-methyl, propoxur and bendiocarb are currently used for IRS in Ethiopia and deltamethrin is incorporated in LLINs. Insecticide impregnated papers were obtained from the WHO Collaboration Centre, Vector Control Research Unit, School of Biological Sciences, Penang, Malaysia. Batches of 20–25 mosquitoes in four replicates were exposed to insecticide impregnated papers for 1 h in WHO test tubes for all bioassays (except for fenitrothion for which there was 2 h exposure) and knockdown was recorded at 10, 15, 20, 30, 40, 50 and 60 min [36]. A control in two replicates, each with equal number of mosquitoes, exposed to papers impregnated with oil was run in parallel. After the exposure period, mosquitoes were transferred into holding tubes and provided with 10% sucrose solution soaked into cotton pads. Mortality was recorded 24 h post-exposure. Mosquitoes, both dead and alive, were individually preserved in Eppendorf tubes over silica-gel for molecular assays.

### DNA extraction

The DNA of individual mosquitoes was extracted using DNAzol reagent (MRCgene, USA) [37]. Extraction of DNA was carried out from 320 surviving mosquitoes (160 DDT survivors and 160 deltamethrin survivors) following WHO bioassays from each study site. Similarly, DNA was extracted from 73 and 64 dead mosquitoes following DDT and deltamethrin bioassays, respectively. Extraction of DNA was also done from 20 bendiocarb and propoxur surviving mosquito specimens and 20 unexposed mosquitoes.

### Molecular identification of *An*. *gambiae* complex and detection of target site mutations

Molecular identification of the *An*. *gambiae* complex was carried out by species-specific polymerase chain reaction (PCR) following an established protocol [38] and detection of the *kdr* allele was carried out using allele-specific PCR [20, 21]. To assess the validity of the *kdr* assays, some specimens were directly sequenced (LGC genomics, Berlin, Germany) and sequenced chromatographs were visually inspected to detect both homozygotes and heterozygotes. The genomic DNA of 20 mosquitoes unexposed to any of the insecticides were pooled and amplified to detect N1575Y mutation [23]. PCR amplicons were sequenced by LGC genomics (Berlin, Germany) and chromatographs were visually inspected to detect the N1575Y mutation (numbering according to *Musca domestica* para sequence GenBank, NCBI). Genomic DNA was amplified from 20 survived mosquitoes following bendiocarb and propoxur bioassays in populations of *An*. *arabiensis* [39] and then the resulting PCR amplicons were sequenced by LGC genomics (Berlin, Germany). Sequencing chromatographs were visually inspected to detect the G119S mutation in mosquito specimens.

### Biochemical assays

Mosquito larvae were collected from a range of breeding sites and reared to adults in field testing rooms (temperature 25 ± 2 °C and relative humidity 80 ± 10%) in all the study sites. Female adult mosquitoes (1–3 day-old) unexposed to insecticides were transported and frozen in a -80 °C freezer in the laboratory. Batches of fifty, 1–3 day old frozen female mosquitoes were individually homogenized to assess levels of carboxylcholinesterases, glutathione S-transferases and cytochrome P450 monooxygenases activities using the acetylcholinesterase, gluthathion S-transferase, protein and TMBZ-peroxidation assays, respectively [40, 41]. In these assays, 25 mosquitoes from Sekoru susceptible *An*. *arabiensis* laboratory strain were used as a control. This susceptible *An*. *arabiensis* strain has been maintained for over 35 years in the WHO Malaria Training Center Insectary, Adama, Central Ethiopia. The strain is susceptible to all the tested insecticides. The colony used in the assay has been maintained at Sekoru Tropical and Infectious Diseases Research Centre (TIDRC) Mosquito Insectary, Jimma University, since 2012.

### Data analysis

Differences in mean mosquito mortality rates were analysed for each insecticide separately by a Kruskal-Wallis test, with study site as factor to assess whether mortality rates differ between the study sites (Additional file 1: Table S1). Mean percentage mosquito mortality was presented with 95% confidence intervals based on the Clopper Pearson method.

Knockdown allele frequencies were determined and compared between surviving and dead mosquitoes following deltamethrin and DDT bioassays using the Mantel-Haenszel-Cochran test, with study site as stratification factor to assess whether there is a difference between the phenotype and genotype resistance over the different populations. Furthermore, a Breslow-day test was employed to assess whether the effect is the same over different populations, i.e. test the interaction between the study sites and the *kdr* allele frequency differences. The levels of enzyme activity were compared between the wild populations of *An*. *arabiensis* and the susceptible *An*. *arabiensis* laboratory strain using a fixed effects model and F-test. Dunnett’s multiple comparison adjustment was employed to compare levels of enzyme activities of the *An*. *arabiensis* populations from different study sites against the susceptible *An*. *arabiensis* laboratory strain. To assess spatial variation, we used the same model to compare the difference among wild populations of *An*. *arabiensis* (excluding the reference strain) and compare the study sites pairwise using Tukey’s multiple comparison. A 5% significance level was used during the analysis. Mosquito susceptibility test raw data set, the program used and output of the analysis are presented in Additional file 1: Table S1, Additional file 2: Table S2, Additional file 3: Table S3.

## Results

### Insecticide susceptibility tests

**Fig. 2:**
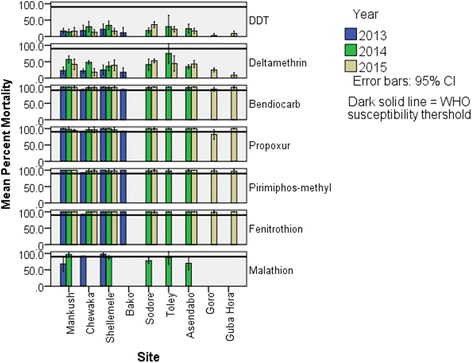
Mean mortality rates of populations of *An*. *arabiensis* exposed to different insecticides. (Dark solid line indicates WHO susceptibility threshold; Error bars 95% CI)

The results of the susceptibility status of populations of *An. arabiensis* from 2013 to 2015 in Ethiopia are presented in Fig. 2. Populations of *An*. *arabiensis* from all sites were resistant to DDT and deltamethrin, according to the WHO criterion. Mean percent mortality rates of mosquito populations of *An*. *arabiensis* against DDT and deltamethrin ranged between 3 and 36% and 9–75%, respectively. The populations of *An*. *arabiensis* from the different study sites were susceptible to fenitrothion. However, few mosquito populations showed reduced susceptibility to malathion, pirimiphos-methyl, propoxur and bendiocarb. Mosquito mortality rates for bendiocarb and propoxur in Goro were 93% and 82%, respectively which, in latter case populations, were resistant to propoxur. Similarly, in 2015 mosquito populations from Mankush, Chewaka and Shellemele showed suspected resistance to propoxur with mortality rates of 94%, 96% and 96%, respectively (Fig. 2). Populations of *An*. *arabiensis* differed significantly for DDT, deltamethrin, bendiocarb and propoxur, whereas no significant difference was observed for fenitrothion, pirimiphos-methyl and malathion (Table 1).

**Table 1:** Mean percentage mosquito mortality rates by population and insecticide

Insecticide	Population	Mean	95% CI		*P*-value
DDT	Mankush	15.7	11.7	20.3	*P* = 0.002
	Chewaka	20.3	15.9	25.3	
	Sodore	27.0	21.0	33.7	
	Asendabo	20.5	15.1	26.8	
	Shellemele	24.0	19.3	29.2	
	Tolay	26.0	20.1	32.7	
	Goro	3.0	0.6	8.5	
	Gubahora	9.0	4.2	16.4	
	Bako	11.0	5.6	18.8	
Deltamethrin	Mankush	40.7	35.1	46.5	*P* < 0.001
	Chewaka	29.2	24.2	34.7	
	Sodore	47.0	39.9	54.2	
	Asendabo	39.0	32.2	46.1	
	Shellemele	33.3	28.0	39.0	
	Tolay	59.4	52.3	66.2	
	Goro	25.0	16.9	34.7	
	Gubahora	9.0	4.2	16.4	
	Bako	18.0	11.0	27.0	
Bendiocarb	Mankush	99.7	98.2	99.9	*P* < 0.001
	Chewaka	96.0	93.1	97.9	
	Sodore	99.0	96.4	99.9	
	Asendabo	99.0	96.4	99.9	
	Shellemele	99.0	97.1	99.8	
	Tolay	100.0	96.4	100.0	
	Goro	93.0	86.1	97.1	
	Gubahora	99.0	94.6	99.9	
	Bako	92.0	84.8	96.5	
Propoxur	Mankush	97.3	94.8	98.8	*P* < 0.001
	Chewaka	95.0	91.9	97.2	
	Sodore	99.5	97.3	100.0	
	Asendabo	100.0	98.2	100.0	
	Shellemele	98.7	96.6	99.6	
	Tolay	100.0	96.4	100.0	
	Goro	82.0	73.1	89.0	
	Gubahora	100.0	96.4	100.0	
	Bako	92.0	84.8	96.5	
Pirimiphos-methyl	Mankush	100.0	98.8	100.0	*P* = 0.058
	Chewaka	96.0	93.1	97.9	
	Sodore	98.5	95.7	99.7	
	Asendabo	99.5	97.3	99.9	
	Shellemele	98.7	96.6	99.6	
	Tolay	98.0	92.9	99.8	
	Goro	98.0	93.0	99.8	
	Guba Hora	97.0	91.5	99.4	
	Bako	100.0	96.4	100.0	
Fenitrothion	Mankush	100.0	98.8	100.0	*P* = 0.062
	Chewaka	97.3	94.8	98.8	
	Sodore	99.5	97.2	100.0	
	Asendabo	100.0	98.2	100.0	
	Shellemele	100.0	98.8	100.0	
	Tolay	100.0	96.4	100.0	
	Goro	99.0	94.6	100.0	
	Guba Hora	100.0	96.4	100.0	
	Bako	100.0	96.4	100.0	
Malathion	Mankush	82.5	76.5	87.5	*P* = 0.056
	Chewaka	92.0	84.8	96.5	
	Sodore	78.0	68.6	85.7	
	Asendabo	70.0	60.0	78.8	
	Shellemele	93.0	88.5	96.1	
	Tolay	88.0	80.4	95.6	
	Goro	94.0	80.0	93.6	

*Abbreviation*: *CI* confidence interval

### Molecular identification of *An*. *gambiae* complex and detection of resistance mutations

Of the 160 *An*. *gambiae* complex samples assayed using species-specific PCR, 159 (99.4%) of the specimens were successfully amplified and all identified as *An*. *arabiensis*. The results of the *kdr* PCR revealed the presence of the *kdr* L1014F allele in all mosquito populations with allele frequency ranging between 42.4–90.6% (Table 2). The *kdr* L1014S allele was absent in all tested mosquito specimens.

**Table 2:** Genotypic and *kdr* allele frequency in populations of *An*. *arabiensis* from Ethiopia

Population	Insecticide	Number assayed	Bioassay phenotype	Genotype			Allele frequency	
				SS	RS	RR	R	S
Chewaka	Deltamethrin	20	Survived	3	6	11	0.70	0.30
		10	Dead	8	1	1	0.15	0.85
	DDT	20	Survived	3	7	10	0.68	0.32
		9	Dead	1	7	1	0.50	0.50
Asendabo	Deltamethrin	20	Survived	0	1	19	0.98	0.02
		9	Dead	5	2	2	0.33	0.67
	DDT	20	Survived	1	5	14	0.83	0.17
		9	Dead	3	4	2	0.44	0.56
Tolay	Deltamethrin	20	Survived	1	0	19	0.95	0.05
		10	Dead	4	3	3	0.45	0.55
	DDT	20	Survived	1	7	12	0.78	0.22
		10	Dead	0	7	3	0.65	0.35
Mankush	Deltamethrin	20	Survived	3	2	15	0.80	0.20
		8	Dead	2	6	0	0.38	0.62
	DDT	19	Survived	2	7	10	0.71	0.29
		9	Dead	0	4	5	0.78	0.22
Shellemele	Deltamethrin	19	Survived	3	4	12	0.74	0.26
		9	Dead	7	2	0	0.11	0.89
	DDT	19	Survived	4	7	8	0.61	0.39
		10	Dead	4	4	2	0.40	0.60
Sodore	Deltamethrin	20	Survived	0	7	13	0.85	0.15
		10	Dead	3	7	0	0.35	0.65
	DDT	19	Survived	5	5	9	0.61	0.39
		5	Dead	0	4	1	0.60	0.40
Goro	Deltamethrin	20	Survived	0	0	20	1	0
		9	Dead	2	4	3	0.56	0.44
	DDT	20	Survived	2	6	12	0.79	0.21
		3	Dead	1	2	0	0.33	0.67
Guba Hora	Deltamethrin	20	Survived	0	0	20	1	0
		8	Dead	0	3	5	0.81	0.19
	DDT	18	Survived	0	0	18	1	0
		9	Dead	1	2	6	0.77	0.23

*Abbreviations*: *SS* homozygous wild type, *RS* heterozygous, *RR* homozygous resistant

Overall, the *kdr* L1014F allele frequency was significantly higher in mosquitoes surviving the deltamethrin exposure, compared to the mosquitoes that died upon exposure (*χ* ^2^ = 126.11, *df* = 1, *P* < 0.0001), and this effect was not differing significantly from population to population (*χ* ^2^ = 8.00, *df* = 7, *P* = 0.3326). Similarly, the *kdr* L1014F allele frequency was significantly higher in mosquitoes surviving the DDT exposure, compared to the mosquitoes that died upon exposure (*χ* ^2^ = 13.10, *df* = 1, *P* < 0.0001) over the different study sites, and this effect was not differing significantly from population to population (*χ* ^2^ = 12.19, *df* = 7, *P* = 0.0945).

The G119S (ace-1^R^ **)** mutation was not detected in mosquito specimens surviving propoxur and bendiocarb exposure. Further sequencing of PCR products of pooled mosquito specimens from each population also confirmed the absence of the ace-1^R^ mutation. Similarly, the N1575Y mutation was not detected in all the assayed mosquito specimens.

### Biochemical assays

The mean percentage of propoxur inhibition in populations of *An*. *arabiensis* ranged from 90.4–94.9% (data not shown here). General esterase assays using α-naphtyl and β-naphtyl acetate as substrates did not reveal elevated levels of esterase activity in all the populations tested, compared to the Sekoru susceptible *An*. *arabiensis* laboratory strain (Table 3). Similar levels of mixed function monooxygenases (MFOs) activities were observed in mosquito samples from all populations, compared to the Sekoru *An*. *arabiensis* laboratory strain. No elevated level of specific esterase activities of pNPA was observed compared to the control. The levels of GSTs activity of the susceptible *An*. *arabiensis* laboratory population were significantly different from the populations of *An*. *arabiensis* from Mankush (*t* = 3.26, *df *= 341, *P* = 0.0064) and Sodore (*t* = 2.88, *df* = 341, *P* = 0.0204). Moreover, there was significant difference in levels of GSTs activities among populations of *An*. *arabiensis* from Asendabo and Mankush (*t* = 3.18, *df* = 320, *P* = 0.0016) (Table 3).

**Table 3:** Levels of esterases (alpha esterases, beta esterases, pNPA), GSTs and MFOs activities (mean ± standard error of the mean) in populations of *An*. *arabiensis* from Ethiopia

	ESTs			GSTs	MFOs
Mosquito population	Alpha naphthyl acetate	Beta naphthyl acetate	pNPA	CDNB	Heme peroxidase
Lab strain	0.024 ± 0.002	0.02 ± 0.008	0.06 ± 0.009	0.023 ± 0.002	0.0012 ± 0.0013
Mankush	0.011 ± 0.005	0.011 ± 0.004	0.049 ± 0.024	0.043 ± 0.005*	0.00092 ± 0.001
Chewaka	0.022 ± 0.008	0.021 ± 0.007	0.067 ± 0.034	0.029 ± 0.002	0.00076 ± 0.0009
Tolay	0.015 ± 0.004	0.012 ± 0.003	0.050 ± 0.09	0.036 ± 0.002	0.00033 ± 0.0004
Asendabo	0.023 ± 0.006	0.022 ± 0.006	0.075 ± 0.036	0.027 ± 0.002	0.00056 ± 0.0006
Shellemele	0.014 ± 0.003	0.013 ± 0.003	0.051 ± 0.027	0.030 ± 0.001	0.00055 ± 0.0006
Goro	0.018 ± 0.008	0.017 ± 0.007	0.06 ± 0.081	0.038 ± 0.005	0.00073 ± 0.0009
Sodore	0.017 ± 0.006	0.015 ± 0.005	0.045 ± 0.047	0.041 ± 0.003*	0.0004 ± 0.0005

* Significant at *P* < 0.05

**Fig. 3:**
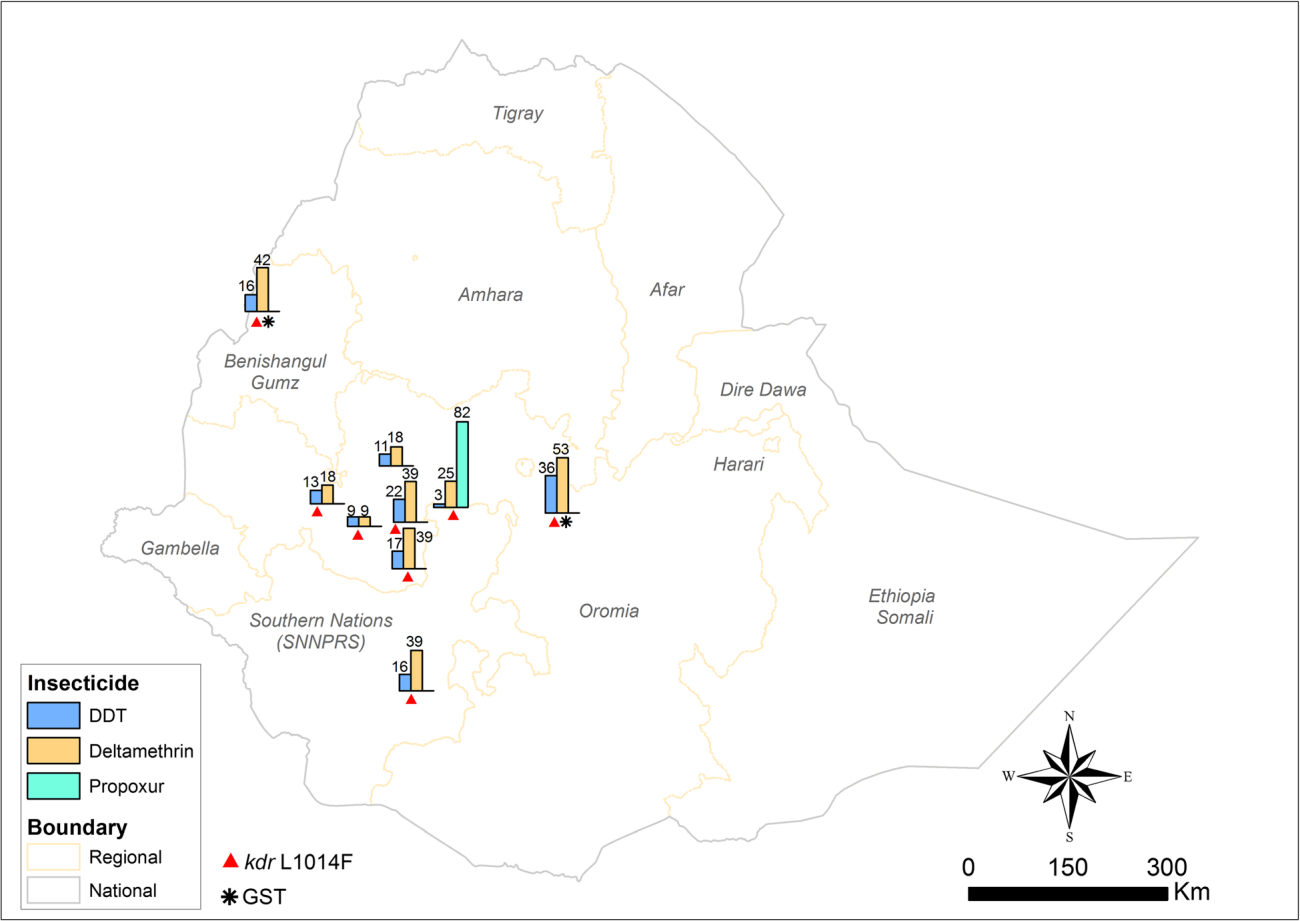
Map showing the overall distribution of insecticide resistance and mechanisms conferring resistance in *An*. *arabiensis* in Ethiopia (Numbers on the bars represent mean percent mortality rates)

Figure 3 presents the overall distribution of insecticide resistance and the underlying resistance mechanisms in the study area. DDT and deltamethrin resistance is widely distributed in populations of *An*. *arabiensis* across the study sites. In contrast propoxur resistance was observed in one locality. There was also widespread of *kdr* L1014F allele. Moreover, elevated levels of GSTs were detected in mosquito populations from two study sites.

## Discussion

*Anopheles arabiensis* was the only member species of the *gambiae* complex recorded from all study areas which is in line with earlier reports from other localities in Ethiopia [12–17]. Previous studies from Gilgel Gibe hydroelectric dam area and other localities in central and western parts of Ethiopia have shown that *An*. *arabiensis* exhibited resistance to DDT and deltamethrin [12–17]. Results from the first insecticide resistance survey (2013) conducted in four study sites and surveys conducted in additional sites from 2014 to 2015 clearly indicated the occurrence of DDT and deltamethrin resistance in this species. This finding was in agreement with the results reported previously from other areas in Ethiopia [12–17] and from many African countries (Chad, Sudan, Tanzania, Uganda and South Africa), where malaria vectors developed resistance to DDT and pyrethroids [9–11, 42–44].

Populations of *An*. *arabiensis* were found to be fully susceptible to bendiocarb, fenitrothion, pirimiphos-methyl and propoxur at most of the surveyed sites. Similar studies from other parts of Ethiopia showed susceptibility of *An*. *arabiensis* populations to these insecticides [14, 17]. Reports from Sudan, Burkina Faso and Chad also showed that *An*. *arabiensis* was susceptible to bendiocarb and fenitrothion [30, 44]. The exhibited resistance in population of *An*. *arabiensis* from Goro to propoxur could threaten the existing vector control interventions by the National Malaria Control Programme (NMCP) of Ethiopia, as propoxur and bendiocarb are currently in use for IRS [34]. Therefore, the emergence of propoxur resistant *An*. *arabiensis* populations is a concern in the use of carbamates for IRS in Ethiopia. The observed resistance to propoxur and suspected resistance to bendiocarb in mosquito populations in Ethiopia calls to implement insecticide resistance management strategy either to delay or slowdown resistance.

DDT and pyrethroid resistance is associated with the presence of *kdr* allele [22]. High frequency of the *kdr* L1014F allele in malaria vectors was first documented and reported some six years back from Gilgel Gibe dam area, southwestern Ethiopia [12]. Later, similar findings were reported from northern, central and south western Ethiopia [16, 17]. The findings of the current study indicated the widespread and high frequency of the *kdr* L1014F allele in many areas. Fixation of this mutation was also recorded in mosquito populations from few localities (Guba Hora and Goro).

The frequency and distribution of ace-1^R^ mutation in *An*. *gambiae* (*s.s*.) has been reported from several African countries [43, 45–47]. The presence of ace-1^R^ mutation in populations of *An*. *arabiensis* was reported for the first time from Burkina Faso, West Africa [43], but this finding has yet to be replicated elsewhere. In the current study, this mutation was not detected by PCR based molecular diagnostics, nor biochemical assays, in mosquito specimens from all sites. The absence of this mutation was also documented in *An*. *arabiensis* from Gilgel Gibe area, southwestern Ethiopia [14]. However, the reduced susceptibility of mosquito populations to propoxur in the absence of ace-1^R^ mutation in few sites warrants further investigation.

In this study, N1575Y mutation was not detected in populations of *An*. *arabiensis* from any of study sites. Similarly, this mutation has not been reported yet from *An*. *arabiensis* [23].

To our knowledge, we report here for the first time a mechanism of metabolic-based resistance operating in populations of *An*. *arabiensis* from Ethiopia. Despite elevated levels of mixed function oxidases and non-specific esterases activities reported in malaria vectors from different African countries [27–29, 47–49], elevated levels of these enzymes were not observed in populations of *An*. *arabiensis* from all study sites. However, studies showed that pre-exposure of mosquitoes to the synergist piperonylbutoxide (PBO) for 1 h before exposure to WHO insecticide impregnated papers increased the susceptibility of *An*. *arabiensis* to deltamethrin [50], which could be attributed to the possible involvement of elevated mixed function oxidases in *An*. *arabiensis*. Interestingly, elevated levels of GSTs were observed in populations of *An*. *arabiensis* from few surveyed sites, suggesting that GSTs might have a role in conferring DDT resistance. Elevated levels of GSTs in *Aedes aegypti* has been reported to confer resistance to DDT [51]. Moreover, upregulation of genes of GSTs in mosquitoes was responsible for DDT metabolism [48, 49]. Therefore, multiple resistance mechanisms (*kdr* L1014F and GSTs) might play a role in the observed resistance in populations of *An*. *arabiensis* to DDT [52–54]*.* The occurrence of elevated levels of GSTs in few mosquito populations could also affect the current use of pirimiphos-methyl for IRS by the NMCP, as cross-resistance between DDT and organophosphate is often caused by GSTs [55, 56]. Furthermore, the involvement of GSTs in mosquitoes may also have implication on the use of organophosphates in insecticide resistance management strategy in Ethiopia.

## Conclusion

Target site resistance due to the *kdr* L1014F allele and metabolic-based resistance due to GSTs appear to be associated with the resistance phenomenon in populations of *An*. *arabiensis* from Ethiopia. The occurrence of GSTs in mosquito populations warrants further investigation as GSTs might confer cross-resistance to many classes of insecticides. The observed elevated levels of GSTs, coupled with high frequency and widely distributed *kdr* L1014F allele in these mosquito populations, could further complicate the current malaria elimination efforts in the country. The reduced susceptibility of some mosquito populations to bendiocarb and propoxur also calls for continuous resistance monitoring, as these insecticides are currently in use for IRS in Ethiopia.

## Additional files

Additional file 1: Table S1. Program and output of statistical analysis. (R 6 kb)

Additional file 2: Table S2. Mosquito mortality data recorded according to WHO insecticide susceptibility test procedure. (CSV 7 kb)

Additional file 3: Table S3. Mosquito mortality data set. (XLSX 21 kb)

## Abbreviations

AChE: Acetylcholinesterases
ASCHI: Acetylthiocholine iodide
CCEs: Carboxylcholinesterases
CDNB: Chlorodinitrobenzene
DDT: Dichlorodiphenyltrichloroethane
DTNB: Dithiobis-2-nitrobenzoic acid
ESTs: Esterases
GSTs: Glutathione S-transferases
IRS: Indoor residual spraying
*kdr*: knockdown resistance
LLINs: Long-lasting insecticidal nets
MFOs: Mixed function oxidases
PBO: Piperonylbutoxide
pNPA: p-nitrophenylacetate
TMBZ: Tetramehyl benzidine
VGSC: Voltage gated sodium channel
